# Metabolic changes in Medaka fish induced by cyanobacterial exposures in mesocosms: an integrative approach combining proteomic and metabolomic analyses

**DOI:** 10.1038/s41598-017-04423-z

**Published:** 2017-06-22

**Authors:** Benoît Sotton, Alain Paris, Séverine Le Manach, Alain Blond, Gérard Lacroix, Alexis Millot, Charlotte Duval, Hélène Huet, Qin Qiao, Sophie Labrut, Giovanni Chiappetta, Joelle Vinh, Arnaud Catherine, Benjamin Marie

**Affiliations:** 1 UMR 7245 MNHN/CNRS Molécules de communication et adaptation des microorganismes, équipe Cyanobactéries, Cyanotoxines et Environnement, Muséum National d’Histoire Naturelle, 12 rue Buffon, F-75231 Paris Cedex 05, France; 2 UMR iEES Paris (CNRS, UPMC, INRA, IRD, AgroParisTech, UPEC), Institute of ecology and environmental sciences - Paris, Université Pierre et Marie Curie, Paris, France; 3 UMS 3194 - CEREEP Ecotron IDF (CNRS, ENS), Saint-Pierre-Lès, Nemours, France; 4 Université Paris-Est, Ecole Nationale Vétérinaire d’Alfort, BioPôle Alfort, F-94704 Maisons-Alfort Cedex, France; 5 ONIRIS, Plateforme de diagnostic et de service d’anatomie pathologie, Ecole Vétérinaire, Agroalimentaire et de l’alimentation, Nantes, France; 6 USR 3149 ESPCI/CNRS SMPB, Laboratory of Biological Mass Spectrometry and Proteomics, ESPCI Paris Tech, PSL Research University, Paris, France

## Abstract

Cyanobacterial blooms pose serious threats to aquatic organisms and strongly impact the functioning of aquatic ecosystems. Due to their ability to produce a wide range of potentially bioactive secondary metabolites, so called cyanotoxins, cyanobacteria have been extensively studied in the past decades. Proteomic and metabolomic analyses provide a unique opportunity to evaluate the global response of hundreds of proteins and metabolites at a glance. In this study, we provide the first combined utilization of these methods targeted to identify the response of fish to bloom-forming cyanobacteria. Medaka fish (*Oryzias latipes*) were exposed for 96 hours either to a MC-producing or to a non-MC-producing strain of *Microcystis aeruginosa* and cellular, proteome and metabolome changes following exposure to cyanobacteria were characterized in the fish livers. The results suggest that a short-term exposure to cyanobacteria, producing or not MCs, induces sex-dependent molecular changes in medaka fish, without causing any cellular alterations. Globally, molecular entities involved in stress response, lipid metabolism and developmental processes exhibit the most contrasted changes following a cyanobacterial exposure. Moreover, it appears that proteomic and metabolomic analyses are useful tools to verify previous information and to additionally bring new horizons concerning molecular effects of cyanobacteria on fish.

## Introduction

Bloom-forming cyanobacteria are ubiquitous organisms of freshwater aquatic ecosystems^1^. To date, mass proliferation of cyanobacteria has been described in numerous lakes and reservoirs, leading to significant health, social and ecological concerns in particular due to their capacity to produce a wide range of bioactive secondary metabolites, called cyanotoxins^2,3^. Among the cyanotoxin diversity, microcystins (MCs) are the most frequent cyanotoxins observed during cyanobacterial blooms of various genera and thus have been largely studied in the past decades. MC-producing and non-MC-producing cyanobacterial genotypes co-occur during blooms in freshwater ecosystems^4,5^. The effects of MC and MC-producing cyanobacteria on various aquatic organisms are being progressively documented^6–9^, in particular on the ichthyofauna which is a relevant indicator of environmental disturbances^10^. MCs are hepatotoxic compounds that accumulate mainly in the fish liver leading to the inhibition of the protein phosphatases 1 (PP-1) and 2 A (PP-2A) and to the occurrence of a cellular oxidative stress *via* the formation of reactive oxygen species (ROS). However, there is still a lack of knowledge concerning the genuine toxicological effects of cyanobacterial blooms themselves, producing or not the MC, especially on fish. Cyanobacteria produce a wide range of secondary metabolites that complicates the decryption and the generalization of these previous experimental observations in an ecological context. Furthermore, the molecular mechanisms managing the differential responses observed in fish and thus explaining the potential deleterious impacts of cyanobacterial blooms on fish populations are still unclear^8,11^. With the development of “Omics” sciences following the analytical progresses of the past decades, transcriptomic, proteomic and metabolomic analyses have proved valuable tools to study an integrated response of an organism in various ecological contexts, allowing the investigation of complex responses of hundreds of transcripts (transcriptome), proteins (proteome) and/or metabolites (metabolome)^12–14^. Although the metabolome is directly influenced by preceding changes in the transcriptome and proteome, it also represents the molecular level at which physiological processes are regulated. While NMR-based metabolomic studies have been widely performed in Human research for drug safety, toxicity assessments, and disease diagnosis^15^, this approach has proved to be very useful to address a wide variety of hypotheses relating to fish physiology and development, pollutant effects and fish condition and disease^16^. However, such investigation has never been applied to evaluate the molecular responses of fish exposed to bloom-forming cyanobacteria, despite it may provide a more comprehensive understanding of what makes cyanobacteria harmful to other living forms.

In this way, a multi-tool approach combining histology, proteomic and metabolomic analyses was performed on males and females of medaka fish (*Oryzias latipes*) exposed for 4 days to environmentally relevant concentrations of a MC-producing (Mcy) or a non-MC-producing bloom (N-mcy) of *Microcystis aeruginosa*. The aim of this study was to investigate and identify early proteinaceous and metabolic changes which may be observed in fish exposed to an MC-producing or a non-MC-producing cyanobacterial bloom in order to support known toxicological knowledge on MC and bring new views about fish-cyanobacteria ecotoxicology in natural environments.

## Results

### Phytoplanktonic and fish experimental parameters

During the experiment, mean total chlorophyll-a (Chl *a*) concentrations (Fig. S1A) reached 138 ± 54 µg.L^−1^, 97 ± 31 µg.L^−1^ and 112 ± 36 µg.L^−1^ for the control, N-mcy and Mcy treatment, respectively. While Chl *a* decreased in both the N-mcy and Mcy treatments, a development of *Scenedesmus sp*. and *Chlorella sp*. was monitored in the control tanks (Fig. S1A,B). In both cyanobacterial treatments (N-mcy and Mcy), a small amount of green algae was detected by the *in*-*situ* fluorometer, corresponding to 15 ± 11% and 14 ± 15% of total phytoplankton biomass in the N-mcy and Mcy treatment, respectively. MC were not detected in both the control and the N-mcy treatments, while total MC concentrations remained relatively stable in the Mcy treatment over the entire course of the experiment (61 ± 8 µg. L^−1^ eq. MC-LR; Fig. S1C). However, intracellular MC concentrations decreased and MC were mainly in the extracellular fraction by the end of the experiment (Fig. S1C). Together with the observed decrease in the cyanobacterial biomass (Fig. S1B), this strongly suggests that bloom was senescent in both cyanobacterial treatments.

No mortality, no abnormality in glycogen storage (PAS) and in liver cell histology (HES) were observed in either male (Fig. S2A) or female medaka (Fig. S2B) exposed for 96 hours to either the green algae control or the MC-producing or non-MC-producing cyanobacterial treatment.

### Chemical screening of cyanobacterial strains

A total of 59 and 41 metabolites were annotated by LC-ESI-Q-TOF-MS and MALDI-TOF, in the MC-producing strain PCC 7820 and the non-MC-producing strain PMC 570.08, respectively (Table 1, Fig. S3). Only one metabolite, acutyphicin, was detected in both strains using LC-ESI-Q-TOF-MS. However, MALDI-TOF analysis suggests that both strains could share 16 other metabolites including aeruginosins (n = 2), anabaenopeptins (n = 2), a radiosumin and 11 uncharacterized metabolites. For the PCC 7820 strains, from the 9 MC variants that were annotated, 3 were detected only by LC-ESI-Q-TOF-MS, 3 other only by MALDI-TOF, while 3 MC variants were detected by both analytical methods. In addition to MCs, other cyanobacterial secondary metabolites including cyanopeptolins (n = 6), microginins (n = 2), aeruginosins (n = 6), cyclamides (n = 5), an aeruginoguanidin, a micropeptin, a mozamide, an oscillatoxin and other uncharacterized metabolites (n = 22) were annotated by LC-ESI-Q-TOF-MS and/or MALDI-TOF. Among the 41 metabolites detected in the non-MC-producing strain PMC 570.08 by LC-ESI-Q-TOF-MS and/or MALDI-TOF, although no MC was observed (Table 1, Fig. S3), a cyanopeptolin, 8 microginins, 4 anabaenopeptins, 2 cryptophycins, a comnostin and various uncharacterized compounds were annotated.

**Table 1:** Chemodiversity of the experimental cyanobacterial strains revealed by LC-ESI-Q-TOF-MS and MALDI-TOF analyses.

Peptide classes	N-mcy (PMC 570.08)	Mcy (PCC 7820 strain)
Uncharacterized metabolites	+(20)	+(22)
Microginins	+(8)	+(2)
Microcystins	−	+(9)
Aeruginosins	+(3)	+(6)
Anabaenopeptins	+(4)	+(3)
Cyanopeptolins	+	+(6)
Cyclamids	−	+(5)
Acutiphycins	+	+
Cryptophycins	+(2)^#^	−
Aeruginoguanidins	−	+^#^
Comnostins	+^#^	−
Micropeptins	−	+^#^
Mozamides	−	+^#^
Oscillatoxins	−	+^#^
Radiosumins	+^#^	+^#^

The signs “+” or “−” refer to the detection or not of the different peptides classes in each strain. The numbers in brackets relate to the number of compounds and/or variants detected for each peptide class in each strain. The sign “#” refer to an annotation performed thanks to molecular mass estimated using MALDI-TOF analysis, without ESI-MS/MS confirmation.

### Proteomic analysis of medaka

**Figure 1:**
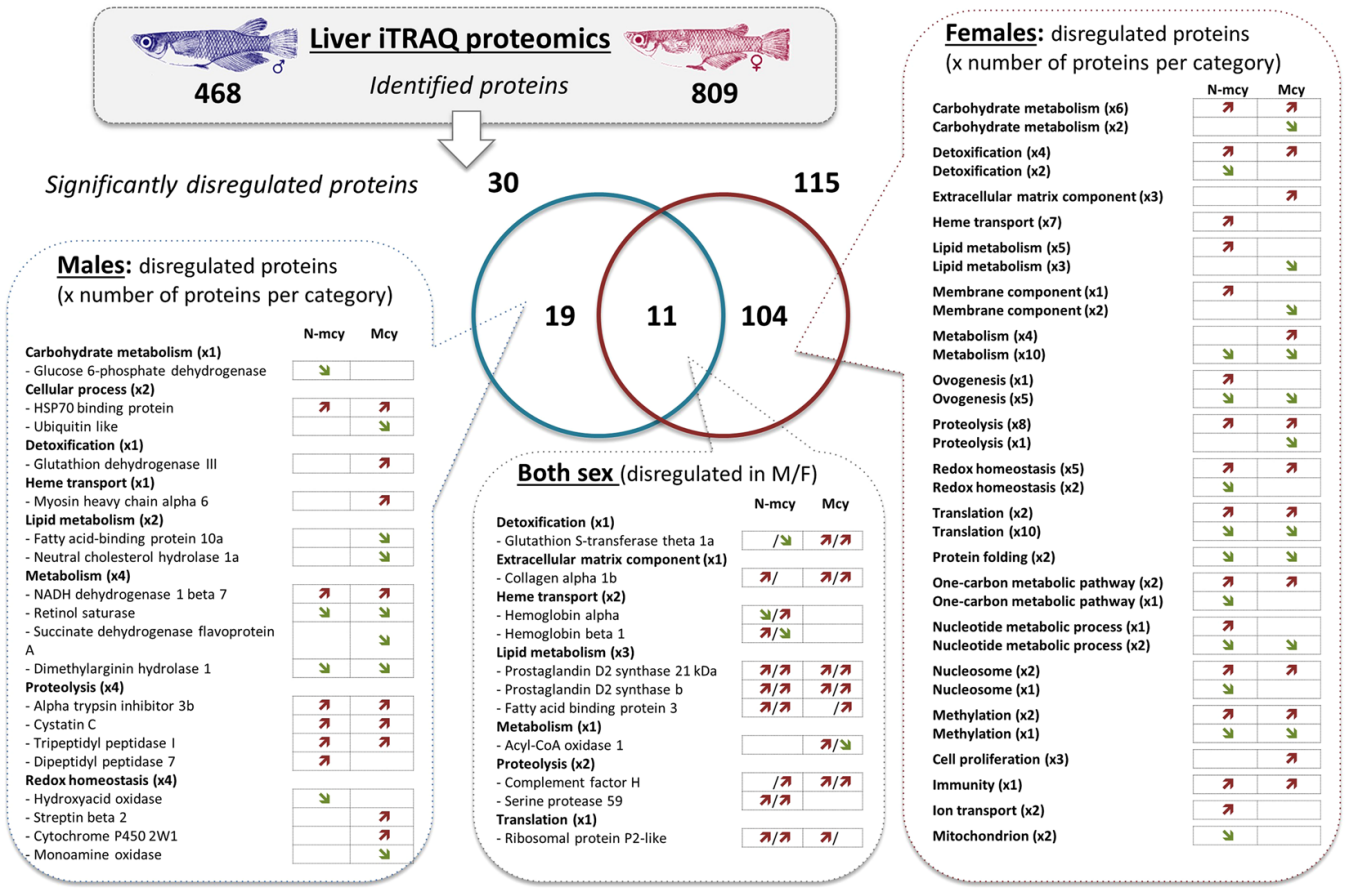
Proteome dysregulations of fish exposed to MCs producing and non-producing strains of cyanobacteria revealed by iTRAQ proteomic analysis. The dysregulated proteins (log2 (|fold-change|) > 0.3 compared to control fish) were shown. The red and green arrows correspond to up and down-regulated proteins respectively.

A total of 468 and 809 proteins were significantly identified in male and female medaka liver, respectively. Quantitative proteomic analysis based on iTRAQ ratios suggested differential expression (*i*.*e*. log2 (|fold-change|) > 0.3) for 134 proteins (Tables S1 and S2). The number of proteins that were specifically dysregulated in females was about 5-fold higher than in males (104 and 19 proteins in females and males, respectively), and 11 common proteins were dysregulated in both males and females but with specific dysregulation patterns according to the gender and the treatment (Fig. 1). Among the dysregulated proteins specifically observed in N-mcy treated fish, 3 proteins implied in carbohydrate metabolism, redox homeostasis, and proteolysis processes were noted in males, while 25 other proteins involved in detoxification, redox homeostasis, one-carbon metabolic pathway, nucleosome, mitochondrion, ion transport, nucleotide metabolic process, oogenesis, membrane components, lipid metabolism and heme transport were observed in females. In addition, 7 commonly dysregulated proteins involved in lipid metabolism, proteolysis, translation and heme transport were observed in both genders of fish exposed to the non-MC-producing cyanobacteria. Following the exposure to MC-producing cyanobacteria, males showed dysregulation of 9 proteins involved in the cellular process, lipid metabolism, metabolism, redox homeostasis, detoxification, heme transport, while in females, 18 proteins involved in carbohydrate metabolism, lipid metabolism, membrane component, proteolysis extracellular matrix component, metabolism, and cellular proliferation were dysregulated. Furthermore, 6 common proteins involved in detoxification, extracellular matrix component, lipid metabolism, proteolysis, and metabolism were dysregulated in both genders treated with the Mcy cyanobacteria. Finally, 7 and 62 other proteins associated with a wide range of biological processes (Tables S1 and S2) were specifically dysregulated in males and females respectively, by both treatments.

In order to summarize the biological sense of the proteinaceous effects of the 2 cyanobacterial strains on male and female medaka, an ingenuity pathway analysis (IPA) was performed. This analysis is based on the fold-change (log2 (|fold-change|) > 0.3) in proteins contents between control (exposure to green algae only) and fish exposed to cyanobacteria (Table 2). It appears clearly that, in both genders, the Mcy treatment induces more protein disturbances than the N-mcy (Table 2). Indeed, fish exposed to this treatment exhibit significant variations in the amount of proteins involved in energy production, lipid metabolism, small molecule biochemistry, molecular transport, free radical scavenging, endocrine system functioning, amino-acid and protein metabolisms and post-translational modifications (−log10 (*p*) > 4) while fish exposed to the N-mcy treatment exhibit significant changes in the amount of proteins involved in energy production, lipid metabolism, small molecule biochemistry and amino acid metabolism (−log10 (*p*) > 4). In addition, a clear difference in these protein content modulations is also observed between males and females, with more proteins dysregulated in females than in males (Table 2).

**Table 2:** Ingenuity pathway analyses (IPA) of medaka exposed to MC-producing and non-producing blooms of *M*.*aeruginosa*.

	Females		Males	
	N-mcy	Mcy	N-mcy	Mcy
Energy production	4.36	10.69	5.99	5.41
Lipid metabolism	5.12	10.69	NS	5.41
Small molecule biochemistry	5.12	10.69	5.80	5.99
Molecular transport	NS	7.28	NS	6.35
Free radical scavenging	NS	6.94	NS	6.68
Endocrine system disorders	NS	6.44	NS	NS
Post-translational modifications	NS	5.23	NS	NS
Protein synthesis	NS	5.23	NS	NS
Amino acid metabolism	5.06	5.13	NS	5.80
Hepatic system disease	NS	8.88	NS	NS
Metabolic disease	NS	8.88	NS	NS

IPA analyses were realized according to proteins log2 (|fold-change|) > 0.3 compared to control. Values correspond to the −log10 (*p*-*value*) > 4 traducing the significance of the dysregulation for each biological processes highlighted. The threshold fixed at 4 corresponds to a *p*-*value* < 0.0001.

#### Metabolomic analysis of medaka

**Figure 2:**
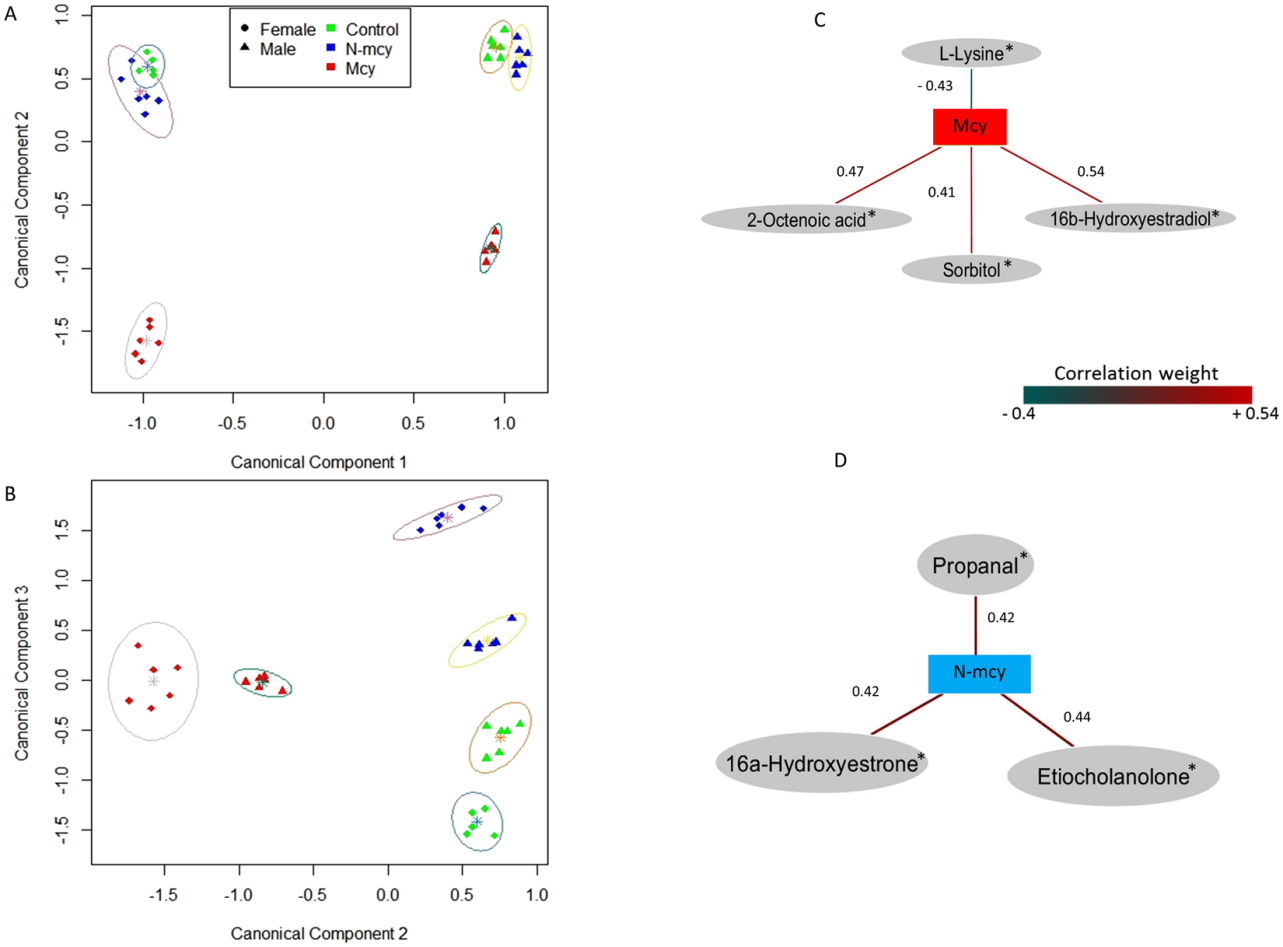
^1^H-NMR liver metabolomes and discriminant metabolites of fish exposed to MC-producing and non-producing cyanobacterial strains. The individual plot of regularized canonical correlations analysis (rCCA) for dimensions 1–2 (**A**) and dimensions 2–3 (**B**). Female-control (green circle), female-N-mcy (blue circle), female-Mcy (red circle), male-control (green triangle), male-N-mcy (blue triangle), male-Mcy (red triangle). Relevance network providing from rCCA analysis on dimension 2 (**C**) and 3 (**D**). Metabolites with a correlation ≥ 0.4 were kept for both treatments. (*) correspond to candidate biomarkers. Red edges correspond to positive correlations and blue edges correspond to negative ones. Values indicated close to the edges correspond to correlation weights between treatments (controlled factors) and every metabolites.

**Figure 3:**
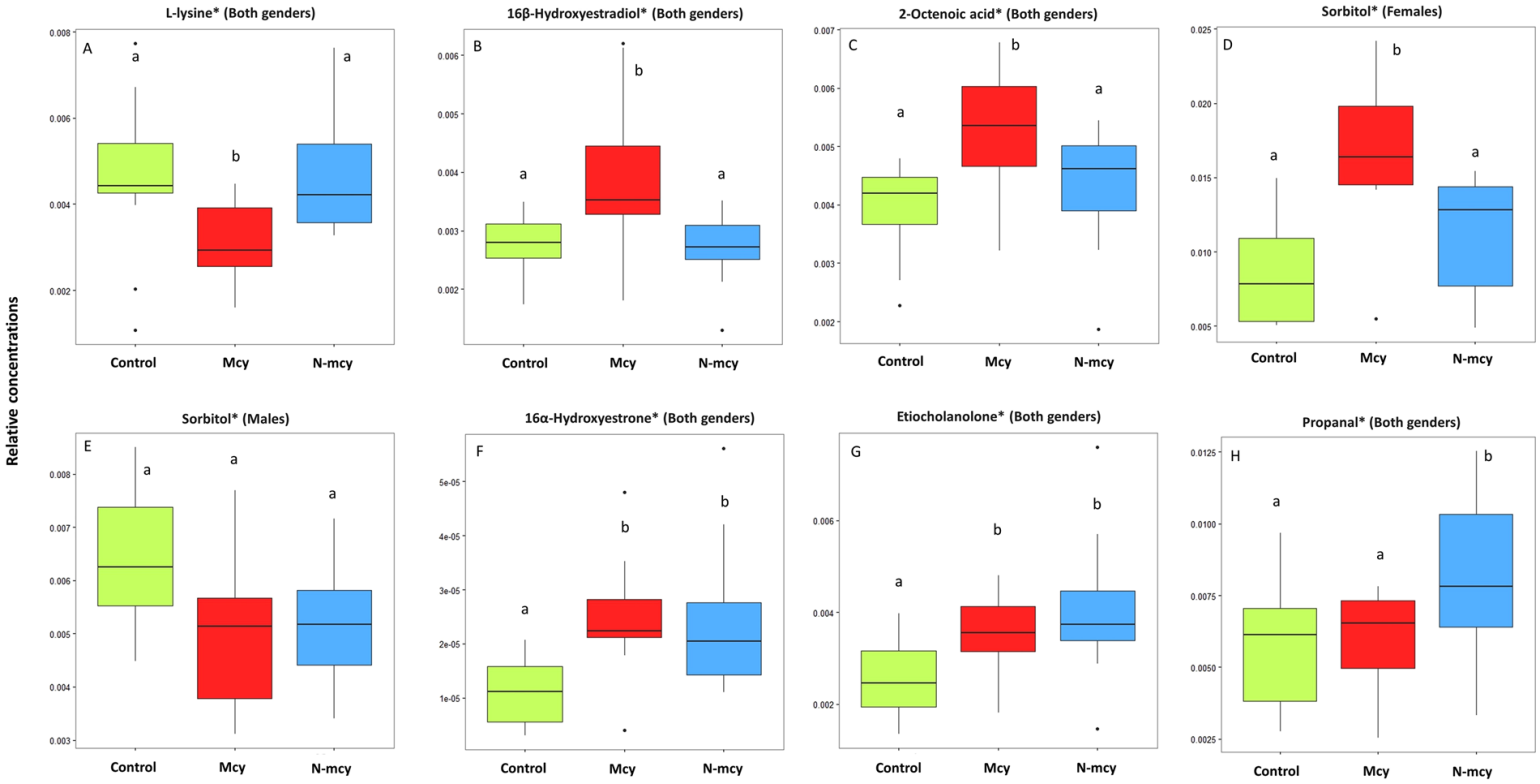
Box plots of the square roots of the relative concentrations of metabolites pointed out by relevance networks. For the metabolite (assigned as sorbitol) showing an interaction effect (gender*treatment), box plots were presented separately for each gender and statistical analyses done separately on each gender. For other metabolites showing no interaction effect, box plots and statistical analyses were done on the complete male and female population. (*) correspond to candidate biomarkers. (**A**) L-lysine*, (**B**) 16β-hydroxyestradiol*, (**C**) 2-octenoic acid*, (**D**) sorbitol* (female), (**E**) sorbitol* (male), (**F**) 16α-hydroxyestone*, (**G**) etiocholanolone* and (**H**) propanal*. Above boxplots, different letters refer to significant differences among treatments (Student-Newman-Keuls post-hoc, *p* < 0.05). Interquartile ranges (25^th^ and 75^th^ percentile) are shown by the height of the boxes, and horizontal lines represent medians (50^th^ percentile). Whiskers range from the 10^th^ to the 90^th^ percentiles, and outside values are indicated by small circles.

Prior to achieving multivariate statistics, ^1^H-NMR raw files were preprocessed thanks to the R “BATMAN” library^17^ to get a relative quantification of a preselected set of analytes. This strategy is only a way to quantitatively document (mean and variance) the presence of these analytes by taking into account in the bayesian calculus the different NMR parameters of these awaited analytes, the final fit resulting into the best quantification relatively to the raw spectrum used. In any case, this procedure can replace an analytical assignment based on different and complementary analytical methods. But because of its intensive nature, this calculus is able to give a confident range around the mean for analytes of a given spectrum, even for very low concentrated analytes when compared to the main ones. Besides, uni- and multivariate procedures used these calculated mean values and can be considered as acceptable given the quality of quantitative data used. For every analyte displaying a significant link to a controlled factor (gender, cyanobacteria exposure, and their interaction), this one can be considered as putative or candidate biomarker. But in absence of confirmatory analytical methods, we prefer to label them using an asterisk showing clearly that they are candidate metabolic biomarkers by sharing with these analytes the same ^1^H-NMR parameters. A multivariate analysis was performed in the “mixOmics” package in R (version 3.2.0) by using regularized canonical correlations analysis (rCCA) between matrix of 225 metabolites (X) and the dummy matrix (Y) corresponding to the controlled factors (gender, treatment, and their interaction). A bootstrap was applied on a MANOVA procedure to the dataset in order to highlight the significant effects of the controlled factors on the observed metabotypes. The MANOVA bootstrap reveals significant effects of gender (*p* < 10^−16^), treatment (*p* < 10^−6^) and the interaction of gender and treatment (*p* < 0.01) from the rCCA model. A clear gender effect is observable (males and females are clearly separated by the dimension 1) whatever the treatment considered (*p* < 10^−16^) (Fig. 2A). Metabotypes linked to the Mcy treatment are also clearly separated on the dimension 2 for both genders (*p* < 10^−16^), with an interaction effect of gender and treatment (*p* < 10^−8^). This is illustrated by greater metabolic differences from the control condition in females than in males (Fig. 2A). The dimension 3 (Fig. 2B) reveals specific metabotypes linked to the N-mcy treatment for both genders (*p* < 10^−16^), with an interaction effect with the gender (*p* < 10^−16^) as illustrated by a stronger dissimilarity between the N-mcy metabotypes and the other treated groups for females. Relevance network based on these second and third rCCA dimensions identifies a strong positive correlation between the dummy variable corresponding to Mcy treated fish group and the metabolites sharing ^1^H-NMR parameters with either 16β-hydroxyestradiol*, sorbitol*, or 2-octenoic acid* for which relative concentrations are increased, and a strong negative correlation with the metabolite sharing ^1^H-NMR parameters with L-lysine* for which relative concentrations are decreased (Fig. 2C). Furthermore, High positive correlations are highlighted between the dummy variable corresponding to N-mcy treated fish group and the metabolites sharing ^1^H-NMR parameters with either 16α-hydroxyestrone*, etiocholanolone* and propanal*, for which relative concentrations were increased (Fig. 2D). Two-way ANOVAs confirm that these metabolites are clearly impacted by the treatment or the gender*treatment interaction (Table 3 and Fig. 3A–H). A significant treatment effect is observed on the relative concentrations of the metabolites sharing ^1^H-NMR parameters with 16β-hydroxyestradiol*, 2-octenoic acid* and L-lysine*. Higher relative concentrations of the metabolites sharing ^1^H-NMR parameters with 16β-hydroxyestradiol* and 2-octenoic acid* (Fig. 3B,C), and lower relative concentrations of the metabolite sharing ^1^H-NMR parameters with L-Lysine* (Fig. 3A) are observed for fish exposed to the Mcy treatment compared to the two other groups (Student-Newman-Keuls post-hoc, *p* < 0.05). Furthermore, a significant gender*treatment interaction effect is observed on the relative concentrations of the metabolite sharing ^1^H-NMR parameters with sorbitol* (Fig. 3D,E) with higher relative concentrations for female fish exposed to the Mcy treatment whereas no significant differences are observed for males (Student-Newman-Keuls post-hoc, *p* < 0.05). Finally, a significant treatment effect is observed on the relative concentrations of the metabolites sharing ^1^H-NMR parameters with 16α-hydroxyestrone*, etiocholanolone* and propanal* (Student-Newman-Keuls post-hoc, *p* < 0.05), and explained by higher relative concentrations of these compounds in fish exposed to the N-mcy treatment (Fig. 3F–H). However, significant differences in the relative concentrations of the metabolites sharing ^1^H-NMR parameters with 16α-hydroxyestrone* and etiocholanolone* are also observed for fish exposed to the Mcy treatment compared to control fish (Fig. 3F,G).

**Table 3:** Effects of experimental factors on the metabolite expressions revealed by two-ways ANOVA analyses. (*) correspond to candidate biomarkers.

Metabolite	Factors								
	Gender			Treatment			Gender*Treatment		
	*df*	*F*	*p*-*value*	*df*	*F*	*p*-*value*	*df*	*F*	*p*-*value*
L-Lysine*	1	1.150	0.29	2	4.224	**0**.**0242**	2	0.904	0.457
Sorbitol*	1	27.033	**0**.**000013**	2	2.279	0.1198	2	4.313	**0**.**0226**
16β-Hydroxyestradiol*	1	0.138	0.71	2	5.734	**0**.**00778**	2	2.052	0.14
2-Octenoic acid*	1	6.166	**0**.**018**	2	6.312	**0**.**0051**	2	0.410	0.66
Etiocholanolone*	1	2.462	0.12	2	6.071	**0**.**00611**	2	2.958	0.06722
16α-Hydroxyestrone*	1	0.416	0.52	2	7.828	**0**.**00184**	2	1.816	0.18
Propanal*	1	3.028	0.0921	2	5.275	**0**.**0109**	2	2.947	0.0678

## Discussion

### Diversity of cyanobacterial metabolites

Cyanobacteria have the ability to produce a wide variety of secondary metabolites, many of which are non-proteinaceous polypeptides^18^. Besides MCs, for which at least 110 variants have been yet characterized^19^, a great diversity of biologically active metabolites was shown to be produced by cyanobacteria^18,20^. Our results based on mass spectrometry analyses clearly illustrate this chemodiversity and the ability of different genotypes to produce different sets of metabolites (including MCs or not). In the MC-producing strain (PCC 7820), 9 MC variants were detected along with a wide range of other already known peptidic metabolites (*e*.*g*. microginins, aeruginosins, anabaenopeptins, cyclamids and cyanopeptolins). In the non-MC-producing strain (PMC 570.08), we found metabolites belonging to the same peptide classes. However, contrary to the MCs-producing strain, PMC 570.08 seems to produce cryptophycins but lacks the ability to produce MCs and cyclamids. Furthermore, while the peptide classes are generally the same between the two strains, most variants detected in this study are specific to a given strain. Our results are in agreement with many studies that highlight the tremendous chemodiversity of *Microcystis* strains^18,21,22^ and with the previous investigation performed on the two strains used in our study^23^. However, the slight differences existing between our study and previous ones working with these two strains are potentially linked to the difference of the sample preparation, as we studied cyanobacterial biomass complexity instead of cyanobacterial extracts, or to the variations of the metabolite variants that can be differentially produced according to physiological or biosynthetic regulations. The toxicological information available for most cyanobacterial peptides remains largely limited. Many of these substances were shown to be biologically active, mainly described as potent protease inhibitors, and may thus significantly impair aquatic organisms exposed to cyanobacteria^18,24,25^. However, aquatic organisms facing cyanobacterial blooms are exposed to complex cocktails of these secondary metabolites, which may induce a biological response quite different from this reported for a single compound tested in the laboratory. To date, biological information concerning fish exposed to cyanobacterial blooms are still scarce and mostly limited to the investigation of the acute effects of MC on specific molecular targets, such as protein phosphatases or detoxification enzymes^26,27^.

### Global molecular changes following cyanobacterial exposures

Overall, after a short-term exposure of medaka fish to a MC-producing or a non-MC-producing cyanobacterial strain, proteomic and metabolomic analyses achieved on liver clearly indicate that more numerous proteins and biological functions are impacted in female medaka than in males, and especially when they are exposed to the MC-producing cyanobacterial strain. However, the non-MC-producing cyanobacterial strain is also responsible for obvious molecular changes, but to a lesser extent. All these results summarize the fact that both cyanobacterial strains are implicated in metabolic dysregulations in medaka fish but with strain-dependent and sex-dependent effects leading to more molecular modifications recorded in female fish exposed to the MCs-producing strain. Although, our results are in agreement with the fact that the MCs-producing cyanobacterial strains might induce molecular alterations in fish, we show, at both proteome and metabolome scales, that non-MCs-producing strains are also implicated in sex-dependent molecular changes that are involved in many primary biological functions. This highlights the fact that MCs are probably not the only secondary metabolites able to induce a metabolic response in fish and opens new questions about the potential ecological impact of non-MC-producing cyanobacteria in aquatic ecosystems, even though exposure to MC-producing cyanobacteria seems to show more severe molecular changes. In a number of published studies, non-MC-producing strains have been described as “non-toxic” although negative impacts have already been described on different life-history traits of several zooplankton species^7^. According to our results, the term “non-toxic” should be used carefully as fish exhibit fully documented proteome and metabolome changes rapidly after being exposed to non-MC-producing cyanobacteria too. However, the lack of any apical effects detected in the histopathological analysis of the experimental fish doesn’t allow us to clearly state whether the molecular changes observed in this study are relied on the toxic effects of cyanobacteria or to the adaption/detoxification mechanisms involved to counteract the potentially deleterious effects of the secondary metabolites. The sex-dependent response mechanisms have already been highlighted and discussed recently in the liver proteins of fish exposed to pure MC-LR^28,29^ or cyanobacterial extracts^23,30^. The present results also support this assumption at the metabolome scale in fish directly exposed to cyanobacterial biomasses. This underlines the importance of considering fish gender in both experimental and field approaches to avoid any false or incomplete conclusions about the molecular responses of organisms submitted to xenobiotics or toxins in the environment.

### Dysregulated metabolic pathways in fish exposed to cyanobacteria

The dysregulated proteins observed in this study are mainly involved in lipid metabolism, redox homeostasis, proteolysis, general and carbohydrates metabolisms, detoxification and heme transport in both genders, and also more specifically in RNA translation and oogenesis processes in female medaka. The similarity in dysregulated protein categories following the exposure to both MC-producing and non-MC-producing cyanobacterial strains, whatever the gender considered, suggests common responses of fish liver under *Microcystis* bloom pressure. The significant dysregulation of the expression of glutathione-S-transferases (GSTs), cytochrome P450 (CYP450) and peroxiredoxin (PRX), which are involved in detoxification processes and redox homeostasis, are observed for fish exposed to both strains with even more contrasted effects in females. Dysregulation of these stress response associated proteins has already been observed in fish exposed to MC or MC-producing cyanobacterial blooms^31–34^ and our results show here that similar stress responses may be induced by non-MC-producing cyanobacteria. Furthermore, Dysregulations of these proteins at the transcriptome level have also been pointed out in various fish species exposed to MCs. The up-regulation of hepatic *CYP3A65* and its receptor *PXR* transcription levels has been reported in zebrafish acutely exposed to crude MCs^35^. And the dysregulation of mRNA expression of *GST* has been found in liver of Nile tilapia (*Oreochromis niloticus*), goldfish (*Carassius auratus* L.) and silver carp (*Hypophthalmichthys molitrix*) administrated with MC-LR^36–38^. MC and the other potentially deleterious metabolites may lead to a high production of intracellular ROS, inducing the oxidation and then the inactivation of various intracellular proteins preventing their activity and/or their folding. In this way, the increase in proteolysis related proteins as those observed in our study could reflect the degradation of such inactive proteins in lysosomes following a cyanobacterial exposure. Furthermore, we found that proteins involved in the lipid metabolism, such as prostaglandin D2 synthases (PTGDs), apolipoproteins (APOE) and fatty acid binding proteins (FABPs) were also strongly dysregulated. These proteins are particularly important in fat cell differentiation and maturation, lipid cell transports and lipid degradations respectively^39^. Dysregulations of these proteins have previously been evidenced in the literature with respect to exposure to MCs alone^32–34^ and have been supposed to be associated with various lipid metabolic stress response. Moreover, at the transcriptome level, the disturbance of lipid metabolism has also been revealed in medaka fish exposed to MC and MC-producing *Microcystis* extracts^40^. Additionally, metabolomic data show that the putative 2-octenoic acid* and propanal*, two molecules involved in lipid metabolism, are positively correlated to the group of fish exposed to the Mcy and the N-mcy treatments, respectively. Finally, in accordance with previous works performed with different MCs exposures^32–34^, dysregulations in proteins involved in the development of gonads and ovogenesis, such as vitellogenins (VTGs), choriogenins (CHGs) and the hydroxysteroid (17-beta) dehydrogenase 4 (17β-HSD4), are also highlighted by our results in female medaka exposed to the MC-producing strain. Interestingly, the 16β-hydroxyestradiol* seems to be highly correlated with the group of fish exposed to the Mcy treatment. However, physiological concentrations of estradiol-17β and its metabolites are far lower than the micromolar range in tissues and plasma of most animal species and for which the ^1^H-NMR direct detection and quantification would not be accessible. So, it is rather surprising that this candidate metabolite arises as a prominent liver biomarker of a cyanobacterial exposure when raw data are only produced by ^1^H-NMR, an analytical technique which is not very sensitive contrarily to mass spectrometry. The quantitative result obtained by an intensive bayesian calculus performed on a ^1^H-NMR spectrum must be only perceived as an assignment of a prior set of ^1^H-NMR signals detected at different chemical shifts to a given candidate metabolite recorded in a database. Therefore, this cannot constitute a definitive analytical proof to ultimately assign a set of ^1^H-NMR signals to a given analyte. Unfortunately, until now, although promising, nobody has routinely used the R library “BATMAN” to preprocess ^1^H-NMR metabolomic data. Therefore, we cannot know whether there is a possible quantitative bias of the set of the most concentrated metabolites present in the biological matrix on the quantitative bayesian estimation of the set of less concentrated metabolites. If there exists, a very bad individual estimation of the relative concentrations of these minor metabolites would probably be achieved. As a consequence, there would be no mean to significantly model the total variance of these metabolites according to a combination of controlled factors, here the gender and the cyanobacterial exposure, excepted by chance. Paradoxically, from present data, it is merely not the case. More, two candidate biomarkers related to the biotransformation of estradiol-17β, the 16α-hydroxyestrone* and the 16β-hydroxyestradiol*, are evidenced by this calculation approach. Thus, it is unlikely that these candidate metabolites were both detected by chance, even though the true structural characterization may differ from the candidate assignments. In all probabilities, the intensive bayesian calculation based on numerous set of constrained parameters for the set of the 225 analytes, notably the chemical shifts and their possible pH-dependent variations defined in an acceptable range, multiplicity, coupling and ratio of ppm signals for the given analytes, is sufficient to fit the total ^1^H-NMR signal and to reduce the spectrum residual. What we can say is that these low concentrated metabolites, which are significantly assigned as putative steroidal biomarkers when referring to a chemical dataset, would share common chemical shifts and their multiplicity with corresponding still unidentified true metabolic biomarkers. These putative metabolites are biotransformed metabolites of estrogens, playing critical roles in the reproduction, growth, and some developmental processes of fish. Thus, our results could suggest that changes in male and female steroidogenesis processes could occur and be directly induced by the exposure to cyanobacteria. Furthermore, this dysregulation could not be linked solely to the presence of MCs as fish exposed to non-MC-producing cyanobacteria also showed increased content in candidate steroidal compounds, such as etiocholanolone* and 16α-hydroxyestrone*. Dysregulation of proteins such as VTG, PTGDS2, and 17β-HSD4, as well as CYP450 2 family members, which are involved in this steroidogenic reaction^41–43^, could support also this hypothesis. Furthermore, in fish exposed to pure MC and *Microcystis* biomasses or extracts, recent transcriptomic evidences of the dysregulation of VTG, CYP450 and 17β-HSD genes as well as nuclear estrogen receptors PPARa/RXRa, TR/RXR, FXR/RXR, PXR/RXR and LXR/RXR seem to confirm the potential reprotoxic effects of cyanotoxins, and particularly MCs, after short and middle term exposures (96 h to 30 days)^28,40,44^. However, further investigations about the characterization of these putative steroid changes and the chemical identification of the real metabolites involved are needed.

### Conclusions and horizons

In the present study, we demonstrate at both metabolome and proteome sides that MC-producing and non-MC-producing *Microcystis* strains induce a sex-dependent response in medaka fish after a short-term exposure mimicking senescent bloom in natural conditions. However, neither mortality nor histological disturbance was observed over the course of the experiment. Thus, fine molecular markers, such as proteins or metabolites, may be more powerful to detect and characterize early signals of cyanobacterial exposures in fish than classical markers (*e*.*g*. histological damages or death), which generally appear later. One can suppose that different toxic compounds should induce distinguishable and specific molecular signatures in fish metabolism that could likewise be used by scientist as valuable markers for predictive investigations of potential environmental issues.

In natural ecosystems, fish may be exposed to cyanobacterial blooms over the entire summer season, or even longer as some cyanobacterial blooms may be perennial^45^. Accordingly, further studies should address the long-term effects of MC-producing and non-MC-producing cyanobacteria in various fish species to identify potential common and/or specific biomarkers of cyanobacteria exposure together with their ecotoxicological effects. Furthermore, further studies should be probably addressed with juveniles of fish for which recruitment rates contributes substantially more to population stability than variation in adult mortality^46^. In addition, as proteome and metabolome are dynamics and interactive processes, further studies integrating time-series and dose-dependent surveys could be recommended to analyze the results from a narrower mechanistic point of view. We suggest that the use of metabolomic analyses, combined as far as possible to other omics technologies, will help to better understand the molecular interactions between fish and cyanobacteria. More generally, metabolomic studies as illustrated here could become an essential and probably a pivotal tool in the near future for environmental risk assessment of pollutants and for environmental monitoring.

## Methods

### Fish rearing and cyanobacteria cultivation

The study was conducted using six-month-old male and female of the inbred cab strain of medaka (average length of 2.8 ± 0.1 cm and 2.9 ± 0.1 cm and an average weight of 0.38 ± 0.04 g and 0.45 ± 0.05 g, respectively). The animals were handled and experiments were performed in accordance with European Union regulations concerning the protection of experimental animals and the experimental procedures were approved (N°68-040 for 2013-18) by the “Cuvier’s ethical committee” of the Muséum national d’Histoire naturelle (French national number C2EA - 68). Mature adult fish were reared in optimal growing conditions^47^ at the laboratory in aerated fresh water tanks, filled with a mixture of dechlorinated tap water and reverse-osmosis filtered water (1/3–2/3 respectively) at 25 °C and with a 16 h/8 h light/dark cycle. Fish were fed three times a day with commercial dry pellets.

The study was carried out with monoclonal cultures of *Microcystis aeruginosa* maintained in the Paris Museum Collection (PMC, http://www.mnhn.fr/fr/collections/ensembles-collections/ressources-biologiques-cellules-vivantes-cryoconservees/microalgues-cyanobacteries). The PCC 7820 strain was selected as a MC-producer (Mcy) and the PMC 570.08 strain as a non-MC-producer (N-mcy)^23^. A large volume of both dense cyanobacterial strains was cultivated in PTFE plastic bags (20 L) at 25 °C using a BG-11 medium with a 16 h: 8 h light/dark cycle (60 µmol.m^−2^.s^−1^) and a constant filtrated air bubbling (0.2 µm, Sartorius Minisart). Prior to the exposition of fish, chlorophyll-*a* equivalent concentrations (µg.L^−1^ eq. Chl *a*) were estimated for each strain using a bench-top fluorometer (Fluoroprobe II, Bbe-Moldenke, Germany).

### Experimental design, exposure and tissue collection

Three exposure conditions were constituted in 9 distinct tanks filled with a tap water dechlorinated beforehand (750 L each, three tanks per condition). The exposure concentrations of algae were chosen to reflect the environmental concentrations measured on peri-urban ponds of the Ile-de-France region^48^, where frequent cyanobacterial blooms are observed. Three tanks contained only green algae (*Scenedesmus sp*. and *Chlorella sp*.) to obtain an average initial concentration of 142.7 ± 10.6 µg.L^−1^ equ. Chl *a* for the control condition (Control). Three tanks were used as a non-MC-producing (N-mcy) treatment with an average initial concentration of 115.1 ± 3.2 µg.L^−1^ eq. Chl *a* of strain PMC 570.08, then three tanks were used as MC-producing (Mcy) treatment with an average initial concentration of 128.0 ± 2.2 µg.L^−1^ eq. Chl *a* of strain PCC 7820.

In each tank, 6 male and 6 female medaka were separately maintained in distinct keepnets (30 L) and acclimatized one week before exposures. Then, fish were exposed to the three experimental treatments for 96 hours, being fed twice a day. At the end of the experiment, fish were euthanized and dissected, two individual fish livers were sampled for metabolomics analysis and two livers for proteomics analysis, samples were directly deep-frozen in liquid nitrogen and stored at −80 °C until further analysis. Finally, the two remaining livers were fixed in formaldehyde 4% (v/v) for anatomo-pathology analyses.

### Physico-chemical and phytoplankton parameters monitoring

At 0, 6, 12, 24, 48, 72 and 96 hours of experiment and in each tank, a bench-top fluorometer (Fluoroprobe II, Bbe-Moldenke, Germany) was used to estimate the chlorophyll-*a* concentrations. In parallel, samples of water were filtered through 1.2 μm GF/C filters (Nucleopore, Whatman) and stored at −80 °C until total chlorophyll-*a* concentrations analyses described by Yepremian *et al*.^49^. Briefly, filters were freeze-thaw to induce cell lysis and 10 mL of 100% ethanol were added. Filters were vigorously shaken and vortexed to break down the filter paper. Then, tubes containing filters were placed for 10 min at 75 °C and sonicated for 10 min in an ultrasonic bath with tap water and ice (this step reduces the turbidity of the extracts). Finally, samples were centrifuged for 10 min, at 10 °C, 4000 rpm (Eppendorf) and the absorbance of supernatants were read at 665 nm. Chlorophyll-*a* concentrations were calculated using the equation proposed by Ritchie^50^.

Homogeneous temperature (T°), pH, dissolved oxygen (DO), nitrite (NO_2_^−^) and nitrate (NO_3_^−^) ions concentrations were measured between tanks during the entire experiment, and the values stayed in the range of those suggested for medaka rearing during toxicological experiments^47^.

Finally, samples of water were filtered through 1.2 μm GF/C filters (Nucleopore, Whatman) and both the filter and the filtrate were kept at −80 °C prior to intracellular and extracellular MC content analyses. For intracellular microcystins extraction, each filter was placed in a glass tube, extracted with 5 ml of 75% methanol and sonicated for 5 min in an ultrasonic bath. This step was performed twice. The crude extract was centrifuged at 20,000 rpm for 15 min at 4 °C. The supernatant was then collected with a Pasteur pipets and then kept at 80 °C prior to MC analysis. For extracellular content analysis, filtrates were centrifuged at 20,000 rpm for 15 min at 4 °C and then analyzed by ELISA. Both intracellular and extracellular MC content analyses were performed by ELISA analysis according to the microcystins (Adda-specific) Kit (Abraxis LLC). Prior to analysis, samples were dissolved with the ELISA sample diluent to reach, for intracellular samples, a methanol concentration below 5% to avoid any matrix effect and to stay in the detection range of the kit (0.1–5 µg.L^−1^) for all samples. The absorbance was measured at 450 nm. The results were expressed as microgram of MC-LR equivalents per liter (µg·L^−1^).

### Mass spectrometry characterization of secondary metabolites detected in the cyanobacterial strains

The biomasses of the two *Microcystis* strains were filtered, and freeze-dried. The lyophilized cells were then sonicated in 80% methanol, centrifuged at 4 °C (4,000 g; 10 min). The supernatant was transferred and acidified with formic acid and 5 µL were analyzed on an HPLC (Ultimate 3000, ThermoFisher Scientific) coupled with a mass spectrometer (ESI-Qq-TOF QSAR Pulsar, Sciex), and MCs were quantified by AD4G2 ELISA tests (Abraxis).

High-performance liquid chromatography (HPLC) of 5 µL of each of the metabolite extracts was performed on a capillary 1 mm-diameter C_18_ column (Discovery® Bio wide pore 5 µm, Sigma) at a 50 µL.min^−1^ flow rate with a gradient of acetonitrile in 0.1% formic acid (10 to 80% in 60 min). The metabolite contents were analyzed with an electrospray ionization hybrid quadrupole time-of-flight (ESI-QqTOF) hybrid mass spectrometer (QStar® Pulsar i, Applied Biosystems®, France) on positive mode using information dependent acquisition (IDA), which allows switching between MS and MS/MS experiments, as previously described^33^. The data was acquired and analyzed with the Analyst QS software (Version1.1). Peak lists were generated from MS/MS spectra acquired between 10 and 55 min, filtering noise threshold at 2% maximal intensity and combining various charge states and related isotopic forms. Metabolite annotation was attempted according to the precise mass of the molecules, then to their respective MS/MS fragmentation pattern with regard with an in-house database of above 600 cyanobacterial metabolites.

Additionally, *Microcystis* extracted metabolites were directly analyzed on MALDI-TOF (Voyager DE – Sciex). Metabolite extracts were diluted (1:1) in CHCA matrix (cyano-4-hydroxycinnamic acid) 70% ACN containing 0.3% TFA, then 200 count spectra were acquired, data treated with MALDIquant R package (Gibb et Strimmer. 2012), and annotation attempted by comparing monoisotopic mass to our in-house cyanobacteria metabolite databases.

### Histopathology

Liver samples were fixed in cold 10% buffered formalin (4 °C, 48 h), then transferred into 70% ethanol, dehydrated in successive baths of ethanol (from 70 to 95%), and then embedded in paraffin. Blocks were cut in 3.5 μm thick sections, and slides were stained with hematoxylin-eosin-saffron (HES) or periodic acid-Schiff (PAS), according to the standard histological procedure.

### Protein extraction and analysis

iTRAQ based quantitative proteomic was performed for each experimental group (Control, Mcy and N-mcy) on 6-pooled livers for each gender as previously described^33^. Six-pooled livers from adult male and female medaka fish were homogenized on ice in 600 µL of lysis buffer containing 8 M urea, 500 mM triethylammonium bicarbonate buffer (TEAB, pH 8.3), 0.1% SDS and 10 µg of protease inhibitor mixture (Roche, Switzerland). The homogenates were centrifuged at 4 °C (12,000 g; 10 min), and then the supernatants were collected. Proteins were precipitated with TCA 20% (w/v) for 30 min at 4 °C. Then, tubes were centrifuged for 5 min (2000 × g; 4 °C) and the pellet was washed with ice-cold acetone and centrifuged for 5 min (2000 × g; 4 °C). The washing step was repeated twice and then the pellet was dried at room temperature before resuspension in the lysis buffer. The protein concentration was measured using a micro-BCA kit (Sigma-Aldrich, USA), with BSA as a protein standard. For each treatment, 100 µg of protein was reduced, alkylated, digested, and labeled with iTRAQ reagents according to the manufacturer’s protocol (Applied Biosystems, France) with some modifications. For tryptic digestion, samples were incubated with trypsin (1:20, w/w) (Sigma–Aldrich, USA) at 37 °C for 16 h. The protein digests (100 µg) obtained from fish control, fish treated with a non-MC-producing cyanobacterium (N-mcy), and fish treated with an MC-producing cyanobacterium (Mcy) in male-treatments were labeled with iTRAQ reagents 117, 119 and 121, respectively. For females, pooled protein digests from control, N-mcy and Mcy were labeled with the iTRAQ reagents 118, 119 and 121, respectively. After 2 h at room temperature, the labeled samples were combined and evaporated. Prior to mass spectrometry analysis, all samples were desalted using Empore C-18 columns (3 M, St. Paul, USA) and dried by vacuum centrifugation. Mass spectrometry analysis of the 8-plex tagged peptide digests was performed with a Q Exactive™ Hybrid Quadrupole-Orbitrap™ mass spectrometer (Thermofisher Scientific). Liver protein digests were concentrated on C18 stages tips, recovered in 40 µL 2% aqueous TFA, 2% ACN before injection in triplicates (6 µL injected). NanoLC was performed on an Ultimate 3000 RSLCnano System (Thermofisher Scientific): digests were desalted on a trap column (Pepmap, C_18_ 300 µm × 5 mm, 5 µm 100 Å, Dionex) with water containing 2% ACN with 0.1% formic acid (solvent A) for 6 min, and the peptides were finally eluted from a separation column (Pepmap, C18 75 µm, × 500 mm, 3 µm 100 Å, Dionex). The separation gradient as optimized for the samples and is divided in 3 successive slopes: 2–20% in 120 min, 20–35% in 45 min and 35–80% ACM + 0.1% formic acid (solvent B) at flow rate of 300 nL.min^−1^. Each MS spectrum acquisition (*m/z* 400–2000, 70,000 Res.) was followed by up to ten data dependent HCD MS/MS spectra (first fixed mass *m/z* 90, 17,500 Res., 30 normalized collision energy) with an isolation window *m/z* 2 and a dynamic exclusion window of 30 s.

All MS/MS-analyzed samples were analyzed using Mascot 2.4.1 (Matrix Science, UK) and Scaffold software (version 4.5.1; Proteome Software, USA) to search Uniprot databases of Teleostei (downloaded in December 2015). The ion mass tolerance and the parent ion tolerance were set to 20 mDa and 10 ppm, respectively. The methyl methanethiosulfonate of cysteine was specified as fixed modifications. Oxidation of methionine and deamination of N and Q were specified as variable modifications. Scaffold was used to probabilistically validate the protein identifications derived from MS/MS sequencing results. Protein quantification was performed with Scaffold Q + (4.5.1) using the isobaric tag peptide and protein identifications. Protein identifications were accepted if they could be established with more than 99.0% probability and contained at least 2 identified peptides that were quantified using the centroid reporter ion peak intensity. Protein quantitative ratios were calculated as the median of all peptide ratios of the three consecutive runs. Quantitative ratios were log2 normalized for final quantitative testing, using control value set up as a reference sample in both sexes.

### Tissue extraction procedure for metabolomic analysis

Liver extraction was carried out using the methanol/chloroform/water (ratio 2/2/1.8) method based on the existing literature^51,52^. Briefly, fresh frozen livers were weighed and then homogenized in the ice cold methanol (8 mL per gram of tissue; AnalaR Normapur, min. 99.8%, VWR, Pennsylvania, USA) and ice cold milliQ water (2.5 ml/g) and then vortexed for 1 min. Subsequently, ice cold chloroform (4 mL.g^−1^; Normapur, 99.3%, VWR, Pennsylvania, USA) and milliQ water (4 mL.g^−1^) were added to extract the hydrophobic metabolites. Then, the mixture was vortexed for 1 min and incubated on ice for 10 min to obtain a complete solvent partition. The resulting supernatant was then centrifuged at 4 °C for 10 min at 2000 g, resulting in a biphasic solution. The upper polar and lower non-polar layers were carefully removed. The upper polar fraction was then transferred to 2 mL Eppendorf tubes, dried under Speed-vac device (Speed-vac Plus SC110A, Savant) and then kept at −80 °C until NMR analysis. Prior to ^1^H-NMR measurement, the polar tissue extracts were dissolved in 550 µL of 0.1 M sodium phosphate buffer prepared in D_2_O (10% v/v) containing 0.25 mM sodium-3-tri-methylsilylpropionate (TMSP) as an internal standard. Finally, the resulting samples were transferred to a 5-mm NMR tube (Norell, France) and analyzed immediately by ^1^H-NMR.

### ^1^H NMR spectroscopy

All NMR data were recorded at 298 K on a 600 MHz Bruker AVANCE III HD spectrometer equipped with a 5-mm TCI CryoProbe (^1^H-^13^C-^15^N) with Z-gradient. One-dimensional ^1^H-NMR spectra were acquired using a standard Bruker noesygppr1d pulse sequence to suppress water resonance. Each spectrum consisted of 512 scans of 32768 data points with a spectral width of 7.2 kHz, a relaxation delay of 3 s and an acquisition time of 2.3 s.

### BATMAN metabolite quantification

The relative metabolite quantification was performed using the “BATMAN” (an acronym for Bayesian AuTomated Metabolite Analyser for NMR spectra) R-package^17^, which deconvolutes peaks from 1-dimensional ^1^H-NMR spectra to automatically assign a chemical shift to specific metabolites from a target list and then estimate their respective concentrations. This can be achieved thanks to an implementation of a bayesian-based model procedure. “BATMAN” uses in a two component joint model resonances of every assigned proton from a list of catalogued metabolites and uncatalogued metabolites and noisy information to finally reconstruct the empirical NMR spectrum. Therefore, 225 metabolites were quantified from Bruker files using the following parameters: i) the chemical shift regions belonging to the two following regions: 0.5 to 4.60 ppm and 5.40 to 10.0 ppm, ii) 400 burn-in iterations, iii) 200 post-burn-in iterations and iv) 5000 iterations for batman rerun. Calculations were performed on an HP Z820 workstation using two 3.30 GHz Intel Xeon^®^ CPU E5 processors and 64 Go RAM by activating 15-parallel threads processing. In the document, metabolites marked with an asterisk (*) are candidate biomarkers which display a significant correlation to a controlled factor (gender, cyanobacterial exposure, or their interaction).

### Statistical exploration of data

The mixOmics library was used to carry out the multivariate analyses. Regularized canonical correlation analysis (rCCA) is a multivariate statistical method used to assess correlations between two multivariate datasets acquired on the same individuals. Here, it was simply used as a factorial discriminant analysis which models the relationships between cyanobacterial exposure (control, Mcy and N-mcy) and metabolites expression levels. The *rcc* function was used to define the canonical correlations and the canonical variates; the *network* function was used to produce the network of interactions. Cross-validation of MANOVA results was obtained thanks to a bootstrap-based procedure by applying a random assignment of any statistical individual to a given group of treatment.

In order to evaluate the effects of the controlled factors on the relative concentrations of metabolites highlighted by multivariate analyses and the subsequent relevant networks, simple two-ways ANOVAs followed by a Student-Newman-Keuls post-hoc test were performed.

### IPA analysis

Molecular pathway was determined for our merged proteome data using the Ingenuity Pathway analysis software (V01-04; Qiagen) with the Human orthologous of medaka proteins available from Ensembl online platform (http://www.ensembl.org), according to specific Ingenuity Knowledge Database, which is a repository of biological interactions and functional annotations, with all default tissue and cell settings and relaxed filters.

## Electronic supplementary material

Supporting information
