# Author Correction: Metabolic changes in Medaka fish induced by cyanobacterial exposures in mesocosms: an integrative approach combining proteomic and metabolomic analyses

**DOI:** 10.1038/s41598-018-20638-0

**Published:** 2018-03-06

**Authors:** Benoît Sotton, Alain Paris, Séverine Le Manach, Alain Blond, Gérard Lacroix, Alexis Millot, Charlotte Duval, Hélène Huet, Qin Qiao, Sophie Labrut, Giovanni Chiappetta, Joelle Vinh, Arnaud Catherine, Benjamin Marie

**Affiliations:** 1 UMR 7245 MNHN/CNRS Molécules de communication et adaptation des microorganismes, équipe Cyanobactéries, Cyanotoxines et Environnement, Muséum National d’Histoire Naturelle, 12 rue Buffon, F-75231 Paris Cedex 05, France; 2 UMR iEES Paris (CNRS, UPMC, INRA, IRD, AgroParisTech, UPEC), Institute of ecology and environmental sciences - Paris, Université Pierre et Marie Curie, Paris, France; 3 UMS 3194 - CEREEP Ecotron IDF (CNRS, ENS), Saint-Pierre-Lès, Nemours, France; 4 Université Paris-Est, Ecole Nationale Vétérinaire d’Alfort, BioPôle Alfort, F-94704 Maisons-Alfort Cedex, France; 5 ONIRIS, Plateforme de diagnostic et de service d’anatomie pathologie, Ecole Vétérinaire, Agroalimentaire et de l’alimentation, Nantes, France; 6 USR 3149 ESPCI/CNRS SMPB, Laboratory of Biological Mass Spectrometry and Proteomics, ESPCI Paris Tech, PSL Research University, Paris, France

Correction to: *Scientific Reports* 10.1038/s41598-017-04423-z, published online 22 June 2017

The original version of this Article contained an error in the spelling of the author Giovanni Chiappetta, which was incorrectly given as Giovanni Chiapetta.

This error has now been corrected in the HTML and PDF versions of the Article, and in the accompanying Supplementary Information document.

