# Supplementary material for: Metabolic changes in Medaka fish induced by cyanobacterial exposures in mesocosms: an integrative approach combining proteomic and metabolomic analyses
Source: Sci Rep. 2017 Jun 22;7:4051. doi: 10.1038/s41598-017-04423-z (PMC5481417; doi:10.1038/s41598-017-04423-z)

Supporting information

Metabolic changes in fish induced by cyanobacterial exposures in mesocosms: an integrative approach combining proteomic and metabolomic analyses.

Benoît Sotton 1*, Alain Paris 1, Séverine Le Manach 1, Alain Blond 1, Gérard Lacroix 2,3, Alexis Millot 3, Charlotte Duval1, Hélène Huet 4, Qin Qiao 1, Sophie Labrut 5, Giovanni Chiappetta 6, Joelle Vinh 6, Arnaud Catherine 1, Benjamin Marie 1

1. UMR 7245 MNHN/CNRS Molécules de communication et adaptation des microorganismes, équipe Cyanobactéries, Cyanotoxines et Environnement, Muséum National d'Histoire Naturelle, 12 rue Buffon, F-75231 Paris Cedex 05, France.
2. UMR iEES Paris (CNRS, UPMC, INRA, IRD, AgroParisTech, UPEC), Institute of ecology and environmental sciences - Paris, Université Pierre et Marie Curie, Paris, France.
3. UMS 3194 - CEREEP Ecotron IDF (CNRS, ENS), Saint-Pierre-Lès-Nemours, France.
4. Université Paris-Est, Ecole Nationale Vétérinaire d'Alfort, BioPôle Alfort, F-94704 Maisons-Alfort Cedex, France.
5. ONIRIS, Plateforme de diagnostic et de service d'anatomie pathologie, Ecole Vétérinaire, Agroalimentaire et de l'alimentation, Nantes, France.
6. USR 3149 ESPCI/CNRS SMPB, Laboratory of Biological Mass Spectrometry and Proteomics, ESPCI Paris Tech, PSL Research University, Paris, France.

2 supplementary tables
3 supplementary figures

Supplementary Table 1: Complete list of proteins that are significantly dysregulated by cyanobacterial treatments compared to control in female livers.

			Log2(Fold-change)	
Name	Function	Accession Number	N-mcy	Mcy
Calmodulin 3b	Calcium binding	I6L4R5	0.3	0.2
Serum lectin isoform 3	Carbohydrate binding	H2LG01	0.6	0.4
Alpha-amylase	Carbohydrate metabolism	H2N0D4	0.2	0.3
Asialoglycoprotein receptor 2-like	Carbohydrate metabolism	H2MXH8	0.0	0.4
Chitinase acidic 1	Carbohydrate metabolism	H2M494	1.3	0.4
Enolase 1a	Carbohydrate metabolism	H2LLZ8	0.3	0.2
Glutamine-fructose-6-P transaminase 1	Carbohydrate metabolism	H2M6R6	-0.3	-0.3
Glycerol-3-phosphate dehydrogenase 1a	Carbohydrate metabolism	H2MAL3	0.2	0.3
Sialic acid acetylesterase	Carbohydrate metabolism	H2MHU5	0.2	-0.3
Scavenger receptor B2	Cell adhesion	H2L3X8	0.5	0.2
N-myc downstream regulated 1a	Cell proliferation	H2LER5	0.1	0.3
Ubiquitin-like enzyme 7	Cellular process	H2LED8	-0.3	-0.2
Glutathione peroxidase 3	Detoxification	M1H6G2	0.3	0.3
Glutathione S-transferase 1.2	Detoxification	H2M5R1	-0.4	-0.1
Glutathione S-transferase 2	Detoxification	H2LN28	0.3	0.3
Glutathione S-transferase kappa 1	Detoxification	H2M9B4	0.0	0.3
Glutathione S-transferase rho	Detoxification	H2MF28	-0.3	-0.1
Glutathione S-transferase theta 1a	Detoxification	H2N2A9	0.0	0.7
Glutathione S-transferase theta 1a	Detoxification	H2MWI7	-0.5	0.3
Collagen alpha 1b	Extracellular matrix component	H2M6N2	0.1	1.9
Collagen type I alpha 2	Extracellular matrix component	H2LHF2	0.2	1.3
Collagen, type I, alpha 1a	Extracellular matrix component	A8QX86	0.0	1.4
Hydroxysteroid (17-beta) dehydrogenase 4	Gonad development	H2LEQ2	0.2	0.3
Heme binding protein 2	Heme transport	H2MFD7	0.4	0.3
Heme binding protein 2-like	Heme transport	H2LNC6	0.4	0.3
Hemoglobin alpha	Heme transport	H2LKL6	0.3	0.2
Hemoglobin alpha 1	Heme transport	H2LD06	0.5	0.2
hemoglobin beta 1	Heme transport	Q8JIM9	0.3	0.0
Hemopexin WP65	Heme transport	Q8JIP9	0.7	0.0
Myoblogin	Heme transport	I1SRL0	2.4	0.5
Myosin heavy chain 6	Heme transport	C6L8J0	1.2	0.0
Myosin light chain 10	Heme transport	B9ZZQ5	1.2	0.0
D-dopachrome tautomerase	Immunity	H2M5M8	0.3	0.4
Transferrin-a	Ion transport	H2MPL0	0.6	0.2
Annexin A1a	Lipid metabolism	O93446	-0.1	-0.3
Apolipoprotein A-Ia	Lipid metabolism	H2MLX9	0.3	0.1
Apolipoprotein A-II	Lipid metabolism	H2MG18	0.4	0.1
Apolipoprotein Bb	Lipid metabolism	H2MBD1	0.4	0.3
Apolipoprotein Eb	Lipid metabolism	H2MG05	0.4	0.4
Fatty acid binding protein 11a	Lipid metabolism	H2M559	-0.4	-0.4
Fatty acid binding protein 3	Lipid metabolism	H2M7N9	0.5	0.4
Fatty acid binding protein 7a	Lipid metabolism	H2ME97	0.0	-0.3
Fatty acid synthase	Lipid metabolism	A0A0F7G9Q9	0.5	0.2
Prostaglandin D2 synthase 21kDa	Lipid metabolism	H2LQH0	1.0	0.5
Prostaglandin D2 synthase b	Lipid metabolism	H2LSA0	0.6	0.3
Signal sequence receptor alpha	Membrane component	H2LDF6	0.3	0.2
Signal sequence receptor delta	Membrane component	H2LS18	-0.1	-0.3
Transmembrane protein 214	Membrane component	H2MTM5	-0.1	-0.3
3-oxoacid CoA transferase 1a	Metabolism	H2LGX0	-0.4	-0.1
Acetyl-CoA acyltransferase 2	Metabolism	H2LBC1	-0.4	-0.3
Aconitate 3-methyltransferase	Metabolism	H2MV50	0.3	0.3
Acyl-CoA dehydrogenase	Metabolism	H2LR71	-0.4	-0.3
Acyl-CoA oxidase 1	Metabolism	H2L5A2	0.1	0.3
Aldehyde dehydrogenase 9A1b	Metabolism	H2M419	-0.3	-0.1
Aspartate dehydrogenase	Metabolism	H2M788	0.0	-0.4
Biliverdin reductase B	Metabolism	H2L8G7	0.0	0.4
Disulfide isomerase family A2	Metabolism	H2LX92	-0.1	0.5
Disulfide isomerase family A4	Metabolism	H2LSQ4	-0.1	-0.3
Phosphohistidine phosphatase-like	Metabolism	H2MQA7	-0.3	-0.1
Retinol binding protein 1a	Metabolism	H2MAD5	0.4	0.4
Retinol dehydrogenase 11-like	Metabolism	H2LY96	-0.3	-0.3
UDP glucuronosyltransferase 1b	Metabolism	H2M746	-0.3	-0.2
UDP glucuronosyltransferase 5c2	Metabolism	H2MD16	-0.4	-0.2
Catechol-O-methyltransferase 1	Methylation	H2LXJ9	0.1	0.3
Glycine N-methyltransferase	Methylation	H2MY01	0.4	0.3
Phosphoethanolamine methyltransferase	Methylation	H2LCT1	-0.5	-0.3
ES1 protein homolog	Mitochodrion	H2M702	-0.4	-0.2
Histone H1	Nucleosome	H2M8F1	0.4	0.3
Histone H1	Nucleosome	H2LU02	0.0	0.3
Nucleic acid binding protein a	Nucleosome	H2M789	-0.3	-0.2
Adenylate kinase 2	Nucleotide metabolic process	H2LT42	-0.2	-0.3
Adenylate kinase 4	Nucleotide metabolic process	H2M295	-0.3	-0.2
Nucleoside diphosphate kinase 2b	Nucleotide metabolic process	H2MW80	0.6	-0.1
Carbonic anhydrase	One-carbon metabolic pathway	H2LWI8	0.4	0.2
Carbonic anhydrase II	One-carbon metabolic pathway	H2MBN7	0.4	0.3
Methionine adenosyltransferase alpha 11b	One-carbon metabolic pathway	H2LPD1	-0.6	-0.2
Choriogenin H	Ovogenesis	Q8JHT8	-0.1	-0.4
Choriogenin L	Ovogenesis	H2M2R4	-0.1	-0.4
Vitelline membrane outer layer 1a	Ovogenesis	H2LLD6	0.5	0.2
Vitellogenin 1 precursor	Ovogenesis	H2LNM8	0.0	-0.5
Vitellogenin 3	Ovogenesis	H2LQ37	0.0	-0.3
Vitellogenin II precursor	Ovogenesis	H2LP13	-0.5	-0.7
Calnexin	Protein folding	H2MWK1	-0.4	-0.5
Sec23 homolog A	Protein folding	H2MDD7	-0.3	-0.3
Alpha-2-macroglobulin 1	Proteolysis	A0A0F7G9U8	0.3	0.0
Coagulation factor XI	Proteolysis	H2MEU2	0.8	0.4
Complement component 1q	Proteolysis	H2MLW8	1.0	0.3
Complement component 3	Proteolysis	H2MVB4	0.4	0.4
Complement component 9	Proteolysis	H2LCR1	0.3	0.2
Complement factor H	Proteolysis	H2MXP3	0.5	0.3
Kallikrein	Proteolysis	H2MEZ4	0.3	0.2
Metallo-endopeptidase M12	Proteolysis	H2L3F5	0.7	0.4
Proteasome beta 3	Proteolysis	H2MZ13	-0.2	-0.3
Serine protease 59	Proteolysis	A4UWM7	0.3	0.2
Serpin peptidase inhibitor A	Proteolysis	H2MMF6	0.7	0.2
Alcohol dehydrogenase 8a	Redox homeostasis	H2MZR4	0.3	0.3
Aldo-keto reductase family 7A3	Redox homeostasis	H2LHN4	0.3	0.5
Cytochrome C oxidase 2	Redox homeostasis	C6L6H1	-0.5	-0.3
Cytochrome c oxidase VIb2	Redox homeostasis	H2LVT0	0.3	0.1
Cytochrome P450 2A like	Redox homeostasis	H2L6R3	-0.4	0.0
Peroxiredoxin 1	Redox homeostasis	H2M443	0.3	0.4
Spectrin beta chain 1	Redox homeostasis	H2MFE5	0.3	0.3
Glutaminyl-tRNA synthetase	Translation	H2M4U8	-0.3	-0.2
Glycyl-tRNA synthetase	Translation	H2M0E5	-0.3	-0.1
Leucyl-tRNA synthetase b	Translation	H2L846	-0.2	-0.3
Nuclear ribonucleoprotein A0	Translation	H2L3F3	0.3	0.3
Ribosomal protein L18a	Translation	H2N0P8	-0.2	-0.3
Ribosomal protein L28	Translation	H2LK88	-0.2	-0.3
Ribosomal protein L3	Translation	H2L7K6	-0.3	-0.2
Ribosomal protein L5	Translation	H2LK51	-0.3	-0.1
Ribosomal protein P2-like	Translation	H2LMY7	0.3	0.0
Ribosomal protein S16	Translation	H2MNR2	-0.2	-0.3
Ribosomal protein S23	Translation	H2MSQ6	-0.4	-0.3
Ribosomal protein S27a	Translation	H2LBD5	0.3	0.3
Ribosomal protein S9	Translation	H2M6L4	-0.4	-0.3

Supplementary Table 2: Complete list of proteins that are significantly dysregulated by cyanobacterial treatments compared to control in male livers.

			Log2(Fold-change)	
Name	Function	Accession Number	N-mcy	Mcy
Glucose-6-phosphate dehydrogenase	Carbohydrate metabolism	H2LRX4	-0.3	-0.1
HSP70 binding protein 21	Cellular process	H2LB83	0.3	0.1
Ubiquitin like	Cellular process	H2TNX7	-0.1	0.3
Glutathione dehydrogenase III	Detoxification	Q6R5I9	0.1	-0.4
Glutathione S-transferase theta 1a	Detoxification	H2MWI7	-0.2	0.4
Collagen alpha 1b	Extracellular matrix component	H2M6N2	1.2	0.6
Myosin heavy chain alpha 6	Heme transport	H2MLX5	-0.2	1.1
Hemoglobin beta 1	Heme transport	Q8JIM9	-0.3	-0.1
Hemoglobin alpha	Heme transport	H2LKL6	-0.4	-0.1
Prostaglandin D2 synthase b	Lipid metabolism	H2LSA0	0.5	0.6
Fatty acid binding protein 3	Lipid metabolism	H2M7N9	0.4	0.2
Prostaglandin D2 synthase 21kDa	Lipid metabolism	H2LQH0	0.3	0.4
Neutral cholesterol ester hydrolase 1a	Lipid metabolism	H2M8K0	-0.2	-0.3
Fatty acid binding protein 10a	Lipid metabolism	A0A0F7G9G7	-0.2	-0.3
NADH dehydrogenase 1 beta 7	Metabolism	H2MIX8	0.3	0.3
Succinate dehydrogenase flavoprotein A	Metabolism	H2LNX5	0.1	-0.3
Acyl-CoA oxidase 1	Metabolism	H2L5A2	-0.1	-0.3
Dimethylarginine dimethylaminohydrolase 1	Metabolism	H2M4Q8	-0.3	-0.3
Retinol saturase	Metabolism	H2LCC0	-0.3	-0.3
Serine protease 59	Proteolysis	A4UWM7	0.7	0.2
Tripeptidyl peptidase I	Proteolysis	H2MLP4	0.3	0.3
Dipeptidyl-peptidase 7	Proteolysis	H2MTU3	0.3	0.2
Alpha-trypsin inhibitor 3b	Proteolysis	H2LAK3	0.3	0.3
Cystatin C	Proteolysis	H2M908	0.3	0.4
Complement factor H	Proteolysis	H2MXP3	0.1	0.3
Spectrin beta 2	Redox homeostasis	H2LT16	0.2	0.7
Cytochrome P450 2W1	Redox homeostasis	H2MLQ7	0.0	0.3
Monoamine oxidase	Redox homeostasis	H2MF43	-0.1	-0.3
Hydroxyacid oxidase	Redox homeostasis	H2MY51	-0.3	0.0
Ribosomal protein P2 like	Translation	H2LMY7	0.4	0.4

Supplementary Figure S1: Monitoring of experimental parameters linked to the presence of cyanobacteria. (A) Total Chlorophyll-*a* concentrations (µg L^{-1}). (B) Estimated cyanobacterial concentrations (µg eq. chl *a* L^{-1}) measured by BBE probe. (C) Intracellular and extracellular microcystins concentrations (µg eq. MC-LR L^{-1}) measured by ELISA in the treatment containing the MC-producing cyanobacterial strain (no MCs were detected in the other treatments). All data are represented as mean ± standard deviation (SD) measured in tanks (n=3/treatment).

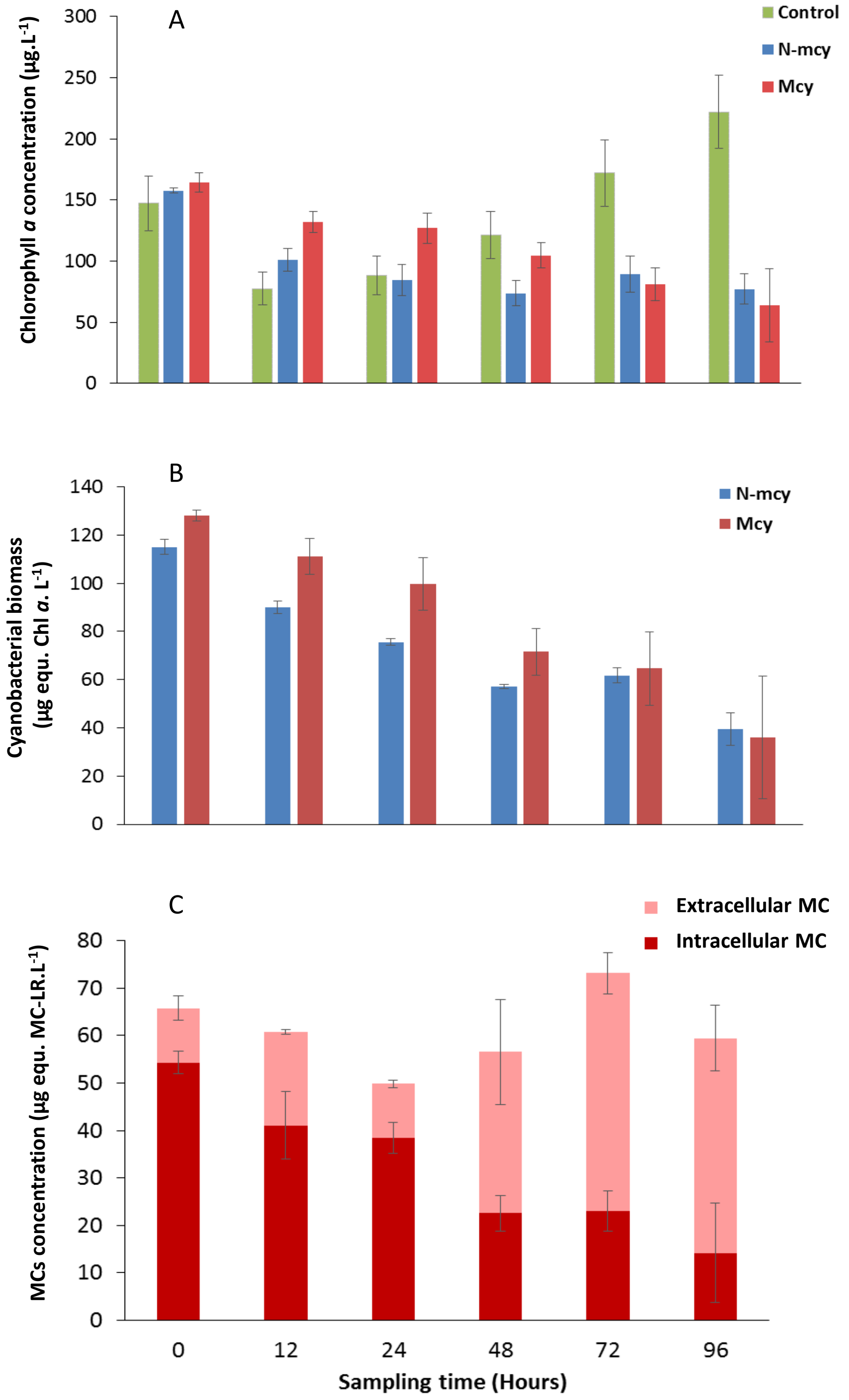

Supplementary figure S2: Light microscopy transverse sections of medaka livers, stained with hematoxylin–eosin–saffron (HES) and Periodic acid–Schiff (PAS). (A) Male and (B) Female medaka.

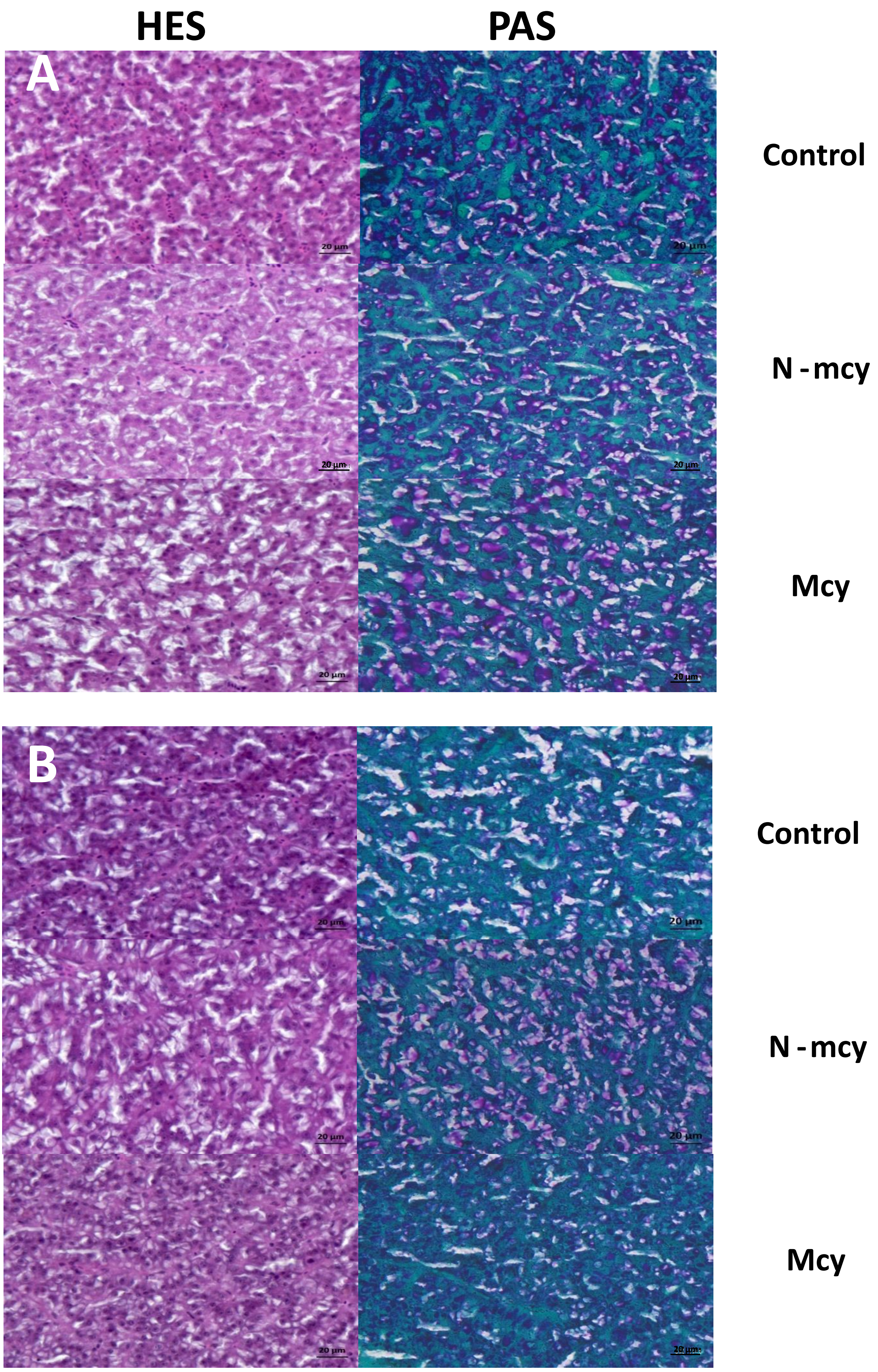

Supplementary figure S3: Venn diagram of the chemodiversity revealed by LC-ESI-Q-TOF-MS and MALDI-TOF in experimental cyanobacterial strains.

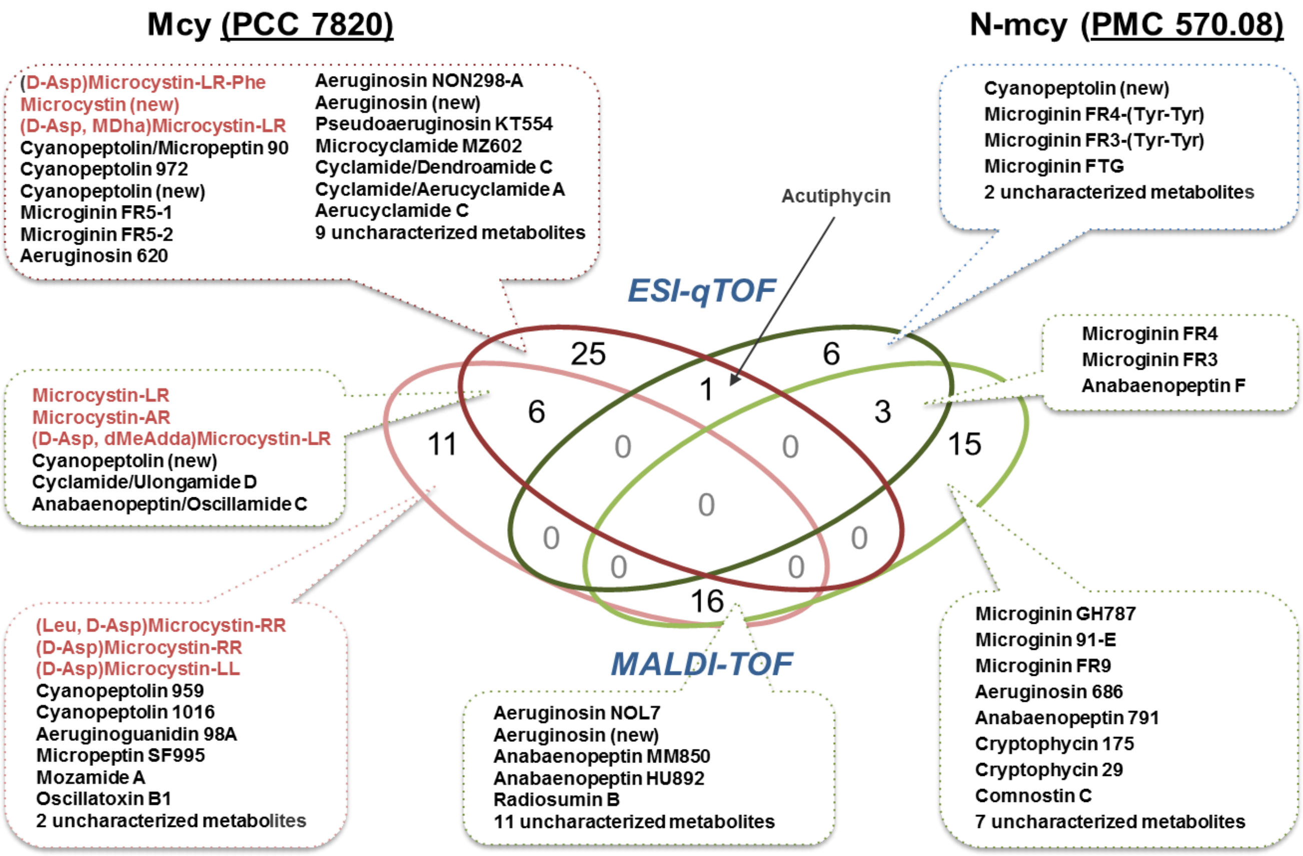

Supplement: Supplementary file 1 — Supporting information [file 41598_2017_4423_MOESM1_ESM.pdf]
